# Application of hyaluronic acid in the healing of non-experimental open wounds: A pilot study on 12 wounds in 10 client-owned dogs

**DOI:** 10.14202/vetworld.2015.1247-1259

**Published:** 2015-10-28

**Authors:** Roberta Ferrari, Patrizia Boracchi, Stefano Romussi, Giuliano Ravasio, Damiano Stefanello

**Affiliations:** 1 Department of Veterinary Science and Public Health, University of Milan, Milan, Italy; 2 Department of Clinical Sciences and Community Health, University of Milan, Milan, Italy

**Keywords:** advance wound dressing, canine, wound assessment

## Abstract

**Aim:**

Veterinarians have frequently to deal with wounds to the skin, subcutis, and underlying muscle. The aim was to explore the application of hyaluronic acid (HA)-containing dressing on open skin wounds in dogs. The progress of healing was assessed by wound area reduction and two scoring scales applied in human medicine.

**Materials and Methods:**

Ten client-owned dogs with 12 cutaneous open wounds healed by the second intention were included. All wounds were treated using available in commerce HA-containing wound dressing from admission to complete re-epithelialization. At every clinical examination, wound area and scale scoring assessments were performed.

**Results:**

After debridement, an increased wound size was obtained while an improvement was determined by both grading systems. The median numbers of return to the clinic for bandage change were 5 times. The median time to complete wound healing was 34.5 days. The mean wound area at day 7, 14, 21, and 28 were, respectively, 90.4%, 47.7%, 22.4%, and 14.8% of the original size (for linear measurement) and 95.5%, 54.4%, 23.10%, and 14.8% of the original size (for software measurement). Regarding wound healing assessment tools, the agreement between two operators was considered high for both scales.

**Conclusions:**

HA-containing dressing may be a possible wound treatment for cutaneous open wounds in dogs. The assessment of wound quality using scale scoring system could be useful especially in the 1^st^ week and to direct clinical decision-making process.

## Introduction

Cutaneous wound is by definition a break or loss of cellular and anatomic continuity of the skin [1]. Many are the possible cause involved in the development of cutaneous wounds in small animal, including bites, car accidents, lacerations from sharp objects, bullets, sticks, burns, and tumor excisions [1,2]. Veterinarians have frequently to deal with wounds to the skin, subcutis, and underlying muscles and even if most wounds heal uneventfully one of the first questions that a clinician should answered is if, when and how close the defect [1,2]. The most satisfactory outcome in wound management is undoubtedly to achieve healing by primary closure [3]. However in many cases, a second intention healing with contraction and epithelialization is required [1,3]. This often happened in presence of large traumatic injuries when there is an abundant amount of devitalized tissue, infection or contamination but also in case of excessive tension on the wound margins after aggressive soft tissue surgery involving skin layers, even as defect leaved by the surgeons or developed as wound healing complication [3-5].

Wound healing is a dynamic process composed by the sequence of different phases: Inflammation, proliferation, and maturation [1]. The management of the wound varies according to wound stage and generally includes irrigation, mechanical, and chemical debridement, adherent and non-adherent dressing and a range of topical medication [6]. Recently in veterinary medicine, many studies have been focused on wound care. In addition, data have been published especially about new healing strategy, techniques, and products such as the use of advance medical dressing and negative pressure therapy with the goal to accelerate the healing process [7-14].

Hyaluronic acid (HA) is a glycosaminoglycan consisted of a basic unit of two sugars, glucoronic and N-acetyl-glucosamine, that forms a part of the extracellular matrix [15]. HA is conserved throughout all mammals and is found in high concentrations in several soft connective tissue including skin [16]. In fact, HA plays a vital role in maintaining tissue integrity, as well as in facilitating adhesion and differentiation of cells during inflammation, wound repair, and embryonic development [17,18]. Many *in-vitro* and *in-vivo* studies confirmed that HA is closely involved in fibroblast proliferation, enhanced formation of granulation tissue and angiogenesis, and even in keratinocyte proliferation and migration during wound healing process [17-21]. These promising results associate with a non-immunogenic response and its degradation by natural, non-inflammatory clearance mechanisms have led to the development of a range of wound dressing containing HA for human medical use [16,22-26]. In veterinary medicine, the information about the use of HA as a part of wound management is little, and nowadays, the benefit results of this molecule application on wound healing as a cross-linked HA-based gel in experimental dog court are still controversial [8,9].

One of the problems encountered in the evaluation of the healing process is also how to evaluate it. The end point of the healing process is to obtain the *restitutio ad integrum* of the skin layer, and the progressive reduction of wound area is the mainstay parameter to assess. Otherwise in recent veterinary studies, the addition of other factors such as characteristics of the wound bed and of the periwound tissue have been discussed as other quality parameters for verifying the healing process and also the wound care therapy application [9,13]. This kind of evaluation using scale scoring system in human medicine, particularly for chronic wounds, is well known even if a gold standard method is not available [27-29].

Due to the little information on literature, the aim of this study was to collate a sample of canine patient with full-thickness skin wounds healed by second intention and to explore on theme the use of available in commerce HA-containing wound dressing, detailing the difficulties, adverse events, costs, and wound outcome. In addition to wound dimensions, the progresses of the wounds were also followed using two wounds assessment scales borrowed from the human medicine that quantitatively described the status of salient physiologic wound characteristics.

## Materials and Methods

### Ethical approval

This study was performed at the Department of Veterinary Science and Public Health of the University of Milan and it did not include experimental animals and it was performed on client-owned dogs referred to our institution for therapeutic purpose. All owners gave us the consensus for treatments, measurements and for data recording. All clinical procedures were performed in according with Italian law (DL 14^th^ march 2014 n.26) and Europe Union legislation covering the use of animals for scientific purpose (“Animal Scientific Procedures Act” 63/2010/EU).

### Inclusion criteria and measurement methods

Animals prospectively included in the study were client-owned dogs with cutaneous open wounds healed completely by only second intention consecutively referred from January 2013 to February 2014. All wounds had at least a full-thickness skin lost and were treated using available in commerce HA-containing wound dressing from the date of admission to the date of complete re-epithelialization. Due to the absence of specific veterinary formulation in our country, HA-based medications available in commerce for human medicine were used, and informed owner consent was obtained for each dog. The complete re-epithelialization was assessed independently by two veterinary surgeons. All animals included had to be followed-up at least until complete re-epithelialization of the wound. Any wound in which was achieved a primary, a delayed primary or the secondary closure were excluded from the study.

All dogs included underwent a physical examination before starting any evaluation and treatment of the wound. For patients referred for traumatic injuries any possible reason of life-threatening were excluded or managed before starting cutaneous open wound evaluation.

Other data recorded in the clinical record were: Signalment, weight and body condition score (BCS - scoring from 1 to 5) [30], etiology (if known), site of the lesion, complete blood sample evaluation (complete blood count, biochemistry), any concomitant drugs administration (e.g. corticosteroids, anti-blastic therapy), and any concomitant disease (e.g. bone fracture, cavitary collections, metabolic, or endocrinologic disease). If referring veterinarian had already treated a wound before referred it, type of treatment and timing of treatment were recorded.

The wound area was calculated using two methods. First, each wound has been approximated to a rectangular figure, and the area was calculated multiply the two maximum perpendicular diameters measured in a bi-dimensional representation. The two measures were achieved using a digital caliper or in presence of wound above the curves of the body whit a soft meter and the limits for the evaluation of the diameters were the macroscopic margins of epithelial tissue. Secondary, a digital photograph of the wound was obtained at each clinical control and uploaded in a wound tracing software program (NIH ImageJ software, http://rsb.info.nih.gov/nih-image/) that calculated the wound area. For both methods, wound area was interpreted as 100% on admission and the areas on subsequent controls were expressed as a percentage of admission value using the formula:

A_t_/A_0_*100

Where, A_0_ is the area on admission and A_t_ is the wound area on control date.

All wounds included in the study were also prospectively classified at admission and during healing process by means of two scale scoring systems, Bates-Jensen Wound Assessment Tool (BWAT) and wound bed scoring (WBS) [28,31-34]. The BWAT includes 4 parameters scoring from 0 to 5 (size, depth, edges, undermining) and 9 parameters scoring from 1 to 5 (necrotic tissue - type and amount, exudate - type and amount, surrounding skin color, peripheral tissue - edema and induration, granulation tissue, epithelialization). The wound assessment results by adding the score of each parameter from a minimum of 9 (the best score) to a maximum of 65 (the worst score) [28,31-33]. The WBS comprised an evaluation of 8 parameters (black eschar - eczema/dermatitis - depth - scarring - color of wound bed - edema/swelling - resurfacing epithelium - exudate amount) scoring from 0 to 2. Using this scale a wound could range from a maximum of 16 (the best score) to a minimum of 0 (the worst score) [34]. In both scales in the presence of multiple features of the same parameter the worst score was reported. Two surgeons independently performed the assessments. The quality and type of exudate were evaluated on the basis of how the removed bandage looked like while all other parameters were assessed after irrigation (with saline) by means of 20 ml syringe and a gentle sterile gauze sponges handing with the purpose to remove the superficial biofilm.

Because wound healing is a dynamic process, all wounds were prospectively managed with HA-containing dressing based on TIME principles [35] in according to the characteristics of the open wound and also to manufacture’s instruction. In presence of necrotic tissue (BWAT amount of necrotic tissue score ≥2 and/or WBS black Eschar score ≤1) a debridement (surgical sharp debridement [SD] and/or medical debridement [MD]) were always applied. The MD consisted of HA plus collagenase-based topical enzymatic debridement agent (Bionect Start^®^, Fidia Farmaceutici S.p.A., Abano Terme - PD, Italy) or a HA plus sodium alginate sterile micro-granules medication (Hyalogran^®^, Fidia Farmaceutici S.p.A., Abano Terme - PD, Italy). In absence of macroscopic necrotic tissue (BWAT amount of necrotic tissue score = 1 and WBS black eschar score = 0) an adsorbent medication made of partial benzyl ester derivative of hyaluronan (Hyalofill-F^®^, Fidia Farmaceutici S.p.A., Abano Terme - PD, Italy) was used when granulation tissue was forming or, when wound did not include deep tissue damage, a daily application of a spray composed by HA plus silver (Hyalosilver^®^, Fidia Farmaceutici S.p.A., Abano Terme - PD, Italy) was applied. When the open wound was entirely filled by granulation tissue (BWAT granulation tissue score = 2 and WBS color of wound bed = 2) the owners were instructed to daily apply a HA based topical medication (Connettivina^®^, Fidia Farmaceutici S.p.A., Abano Terme - PD, Italy) until complete epithelialization. If different and simultaneous features characterized different part of the wound a mix of this product was used. In all cases, the second layer of the bandage was composed by cotton padding and then a third layer of the latex-coated cohesive bandage (Easifix Cohesive^®^, BSN medical, Vibraye, France) was applied. A tie-over bandages was used to fix the second layer in area difficult to cover (e.g. inguinal area).

First wound area and assessments evaluation were performed the date of the first clinical examination for traumatic and primary closure complication wounds (CW), or the date of surgery for excision wounds. After, the assessments and wound area measurement were repeated at every bandage change performed by clinicians. All the bandage changes were sterilely performed. If needed or require by the owner adjunctive control respect to the clinical examination scheduled were performed, and the cause of earlier evaluation was recorded. Numbers and frequency of wound assessment for each animal were also reported. In the case of surgical SD all the assessments, including wound area measurements, were performed both before and after to evaluate the impact of the surgical procedure. When owners applied the HA-medications at home, a weekly clinical examination to assess the wound progress was scheduled. During each clinical examination, owners were asked to inform of any difficulties or disadvantage retrieved with the bandage. The presence of complete wound healing was assessed by the evaluation of the clinician and corresponded to a wound covered entirely by epithelial tissue in the absence of any sign of granulation tissue. Time of complete wound healing (TWH) was recorded and defines as the time from first assessment to complete re-epithelialization (100% of the wound area).

When complete re-epithelialization was assessed cosmetic and functional outcome were also evaluated and recorded in the clinical record.

During the study period, the use of antibiotic therapy was recorded. The use of antibiotics was reserved to wounds showed sign indicative of infection such as: Severe soft tissue swelling, erythema, pain, hyperthermia and/or purulent discharge. The antibiotics were chosen on the basis of anti-biogram results if a swab sample or a biopsy of the wound bed for microbiological culture was available. The antibiotic administration was stopped when the signs of infection were completely regressed, and a healthy granulation tissue (BWAT granulation tissue score = 2 and WBS color of wound bed = 2) was forming.

When the animals underwent general anesthesia, anesthetic protocol consisted of a multimodal approach including analgesic therapy. The protocol differed among animals, and it was calibrated on the basis of the clinical examination of the single animal and the type of procedure that had to be performed (surgical SD vs. bandage change). In all cases after surgical debridement and in every case in which pain was detected an analgesic therapy consisted of non-steroidal anti-inflammatory drug oral administration was prescribed to obtain pain relief.

### Statistical anlaysis

Pattern of wound area reduction (both methods) during follow-up was evaluated by analysis of variance model (ANOVA) for repeated measures. As a pre-fixed schedule for clinical examination was not planned and follow-up time was heterogeneous, 4 times which were common for all subjects were considered: 7, 14, 21, 28 days. To account for the correlation of the measures within the same subject, a variable identifying subject was considered in the ANOVA model as random effect, whereas days was included as fixed effect by dummy variables (mixed model ANOVA). To compare the pattern of the WBS and BWAT with the pattern of wound area reduction the first two scales were transformed as percentages of scoring as follows. For BWAT, the score at time 0 was considered as 100%. The maximum reduction for the patient was the difference between the initial score and the best achieved score (MR BWAT: BWAT at time 0-9). For each time, the difference between the initial score and observed score was calculated (difference BWAT). The percentage was then obtained as:

 100-(diff BWAT/MR BWAT)*100

For WBS, the score at time 0 was considered as 100%. The maximum increase for the patient was the difference between the best score achieved and the initial score (MI WBS: 16-WBS at time 0). For each time, the difference between the observed score and the initial score was calculated (difference WBS). The percentage was then obtained as:

 100-(diff WBS/MI WBS)*100

The pattern of the transformed scales was then analyzed by mixed model ANOVA. The probability of reaching 0% (the best achieved score) during time for wound area reduction, BWAT, and WBS scores was estimated by Kaplan–Meier method. This method allowed to estimate the median time to complete wound area reduction (and the best achieved score of BWAT and WBS). Due to the low number of patients and preliminary aim of this study no formal statistical test on ANOVA were performed. Results are reported as model estimated reduction and 95% confidence intervals (CI). No adjustment was performed by covariate effects.

The agreement between the BWAT and WBS scoring independently attributed to the patients by two veterinary surgeons was evaluated by the intraclass correlation coefficient (ICC) based on two-way repeated ANOVA model with random effects.

As two methods were used to evaluate wound area (the approximation to a rectangular and software), an explorative comparison was performed on all the available wound measurements. No one of the two methods could be considered as gold standard thus the “exact” wound area was approximate by the average of the two calculated areas. The comparison was then examined by plotting the differences between the two areas measures against their average, as suggested in the Bland and Altman plot. Since the aim was only explorative no limits of agreement were calculated but, following a practical perspective, the percentages of measurements differences within defined ranges were reported.

## Results

12 open wounds in ten dogs were enrolled in the study. Breeds were as follows: Mixed-breed (2), Jack Russel Terrier (2), Dogue de Bordeaux (1), Irish Wolfhound (1), Dogo Argentino (1), Whippet (1), Pinscher (1) and American Pit Bull Terrier (1). Four dogs were females (1 neutered) and 6 were males (2 neutered). At admission, mean age was 7.9 years (range 1-15 years) and mean body weight was 23.1 kg (range 5-47 kg). Two dogs had a BCS of 2, in 5 dogs the BCS was 3 and in the last 2 dogs was 4.

At admission, three dogs reported comorbidities related to past history: Cardiopathy (2) and diabetes mellitus (1). Two dogs reported in the recent clinical history (2 weeks or less before admission) one or more surgical malignant tumor excision in location not associated to the wound: Perivascular wall tumor (1), mast cell tumor (1) and femoral osteosarcoma (1). One dog was surgically treated for a foreign body abscess of the mandibular region 2 weeks before admission.

The hematological parameters at admission were unremarkable in all but two dogs in which hypoalbuminemia (1) and high glucose level (1, the dog with diabetes mellitus) were found.

### Wound data

A total of 12 wounds were included (2 dogs had 2 simultaneous and anatomically split wounds) and treated with HA-based medications until complete re-epithelialization. All wounds were summarized in Table-1. The wounds were classified on the basis of the etiology in: Traumatic wounds (TW, 7), primary closure CW (2) or excised wounds (3) in presence of skin defect intentionally leaved by surgeon. The specific nature of trauma was reported in Table-1. Two TW were referred in presence of unhealthy and deteriorating wound appearance after 10 days of previously treatments: Surgical apposition of traumatized cutaneous margins (Table-1) and naftalina granules and saline solution irrigation (Table-1). Regarding wounds developed after malignant tumor excision, the histologic margins were infiltrated in two cases (Table-1). No one wounds presented healthy granulation tissue at time to admission.

**Table-1:** Wounds included in the study.

Wound	Weight (kg) [BCS]	Age (years)	Type	Cause	Site	Area (cm^2^)	TWH (days)	Concomitant drugs
1	38 [4]	5	TW	Laceration (unknown origin)	Elbow	18	24	-
2	15 [2]	1	TW	Hot water pad burn	Medial thigh	9.6	21	-
3	35 [3]	4	TW	Laceration (unknown origin)	Distal forearm	36	62	-
4	21.7 [4]	6	TW	Car accident (degloving injuries)	Ventral abdomen	540	75	-
5	21.7 [4]	6	TW	Car accident (shear injuries)	Lombar region	38.7	35	-
6	9 [4]	8	TW	Electric pad burn	Flank	109.6	54	-
7	9 [4]	8	TW	Electric pad burn	Scapular	22.5	17	-
8	45 [3]	4	EW	Malignant skin neoplasia excision	Lateral hock	36	49	Vinblastin+ corticosteroid
9	5 [3]	14	EW	Malignant skin neoplasia excision	Proximal medial forearm	15.1	28	ACE-inhibitor+ furosemide
10	6 [3]	15	EW	Malignant skin neoplasia excision	Caudal thigh	5.4	34	ACE-inhibitor+ furosemide
11	47 [3]	11	CW	Malignant skin neoplasia excision	Lateral thorax	32	88	-
12	9.2 [2]	11	CW	Forelimb amputation for malignant skin neoplasia	Shoulder	10.4	21	Insulin

TWH=Time to complete wound healing, BCS=Body condition score, TW=Traumatic wounds, EW=Excised wounds, CW=Complication wounds

At admission, the median wound area using the approximation to a rectangular figure was 32 cm^2^ (min 5.40, max 561). In all cases were debridement (SD and/or MD) was applied the wound areas had an average increase of 14.4 cm^2^ (Table-2).

**Table-2:** Wounds received debridement.

Wound	Type of debridement	Two-axis area (cm^2^) at admission	Two-axis area (cm^2^) post-debridement	Software area (cm^2^) at admission	Software area (cm^2^) post-debridment	BWAT at admission	BWAT post-debridement	WBS at admission	WBS post-debridement
2	MD	9.6	19.2	2.67	8.74	33	28	7	12
3	SD	36	51.5	30.43	45.47	46	36	4	6
4	SD	540	561	401.65	417.29	55	45	3	9
5	MD	38.7	58.8	22.24	54.36	32	27	8	11
6	SD	109.6	139.5	4.8	5.43	38	32	11	12
7	SD	22.5	27.3	91.18	91.83	34	24	12	14
12	SD	10.4	11.2	17.83	18.99	38	34	10	11

The assessment for wound underwent only medical debridement referred to day 4 when both the wounds achieved the widest area, for wound underwent surgical debridement referred to day 0 (when the surgery was performed). MD=Medical debridement, SD=Surgical debridement. BWAT=BatesJensen Wound Assessment Tool, WBS=Wound bed scoring

At admission, the median wound area using the software was 22 cm^2^ (min 2.67, max 417.29). In all cases were debridement (SD and/or MD) was applied the wound areas had an average increase of 10.19 cm^2^ (Table-2).

At admission, the median BWAT score was 32.5, while for WBS was 11. In the 7 wounds in which debridement had been applied, the median BWAT score improved from 38 to 32, corresponding to a median decrease from admission score of 6, while the median WBS score improved from 8 to 11, corresponding to a median increase from admission WBS score of 2 (Table-2).

**Figure-1:**
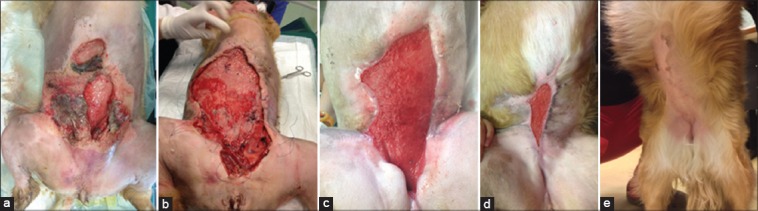
Wound healing evolution for wound 4, (a) Wound status at admission: A wide area of discolored tissue was present on the ventral side of the abdomen. The mammary tissue was mobile respect to the underlying abdominal wall. The patient underwent a massive surgical debridement in association with medical debridement, (b) wound status after 2 days: An initial deposition of granulation tissue was present. Multiple, loose, simple interrupted, monofilament, nonabsorbable suture were used to secure in place the medication and the bandages (tie-over bandage), (c) wound status after 19 days: A decrease of wound dimension was easily appreciated. All of the wound boundaries were fixed to the underlying tissue and at the wound margins a new epidermal edge was visible, (d) wound status at 55 days: A constant decreased of wound area was retrieved. The owners were instructed on the daily application of the topical medication (hyaluronic acid-based cream), (e) wound status at 75 days: Complete epithelialization.

**Figure-2:**
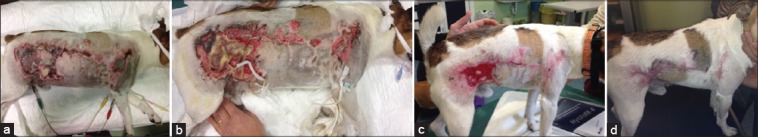
Wound healing evolution for Wound 6 and 7, (a) Wound status at admission: Two areas of discolored tissue were present on the right lateral side of the trunk (wound 6 on the flank and wound 7 on the scapular region). The two wounds were filled by a dry black and yellow eschar. Both wounds were treated with medical debridement. For wound 6 a tie-over bandages was applied, (b) wound status after 4 days: The eschars became an easy to remove yellow non-fixed mushy necrotic tissue. An initial pink granulation tissue was present under the necrotic tissue. Multiple, loose, simple interrupted, monofilament, non-absorbable suture were used to secure in place the medication and the bandages for the wound on the flank (tie-over bandage), (c) wound status after 15 days: A decrease of wound dimension was easily appreciated. Wound 7 was almost complete epithelialized. The bottom of the wound 6 was filled by a healthy red granulation tissue, (d) wound status at 54 days: A complete epithelialization of the wound 6 was achieved.

All dogs were discharged at home the day of admission and returned to the clinic only for dressing changes and clinical controls. The median numbers of return to the clinic for bandage change (excluding the date of admission) was 5 times, (range, 4-14 times). When dressing was applied by the owner wounds were generally rechecked once a week (median, 7 days; range, 2-16 days). When the adsorbent medication made of partial benzyl ester derivative of hyaluronan was applied, the dressing was always revised by the clinician, and bandages were generally changed every 4 days (median, 4 days; range, 2-7 days). In the 2 widest TW (Table-1 and Figures-1 and -2) due to the amount of exudate a silver-pad foam (Cellosorb Silver^®^, Laboratoires URGO, ChenO, Cedex, France) was applied over the HA-based medication before the second layer of the bandage until scoring for the amount of exudate achieved 2 out of 5 for BWAT and 2 out 2 for WBS. Due to the location and the size of the wounds a tie-over bandage was applied in both cases.

In 5 wounds (3 dogs) antibiotic therapy was administered. In wounds 3, 4, 5 (Table-1) a swab test was performed due to the presence of purulent discharged and peripheral tissue erythema. In these cases *Klebsiella pneumoniae* was retrieved and an antibiotic protocol with marbofloxacin was started as anti-biogram results suggested. In wounds 6 and 7, the dog had been already under enrofloxacin administration before the admission, and the protocol was continued until appearance of granulation tissue due to the nature of the lesion even if no particular signs of infection were presented at that moment.

Excluding the SD that was always performed under general anesthesia, only for the management of wound 4 (Table-1) sedation was performed at every bandage change (every 3 days) until day 10 from admission. This was the widest wound and for clinical control the dorsal recumbency was necessary creating particular discomfort for the dog during this initial period. All other bandage changes in this dog and all bandage changes in the other patients were performed with wakeful dogs.

All the products were well tolerated by dogs, and no side effect was retrieved. Only one dog was prone to removing the bandage, and earlier returns to the clinic than scheduled were necessary also because the owner did not follow the suggestion to use Elizabethan collar (Table-1). Some owners complained the presence of an unpleasant odor of the bandage. This was correlated to high amount of exudate and breaks down of the HA-based products but not to infection as also assessed by the improvement of the wound status. When spray formulation was used, owners reported that some dogs felt a little uncomfortable sensation due to the noise. The cosmetic result was considered good even if in all cases an alopecia area of different dimension has been retrieved. In addition, the skin in this area looks thinner and in some cases hyper-pigmented but no complication was reported related to these condition. No range motion alteration due to contracture were observed even for wounds located close to flank, axillar or other joint, and the functional outcome was judged great in all dogs.

### Outcome

**Figure-3:**
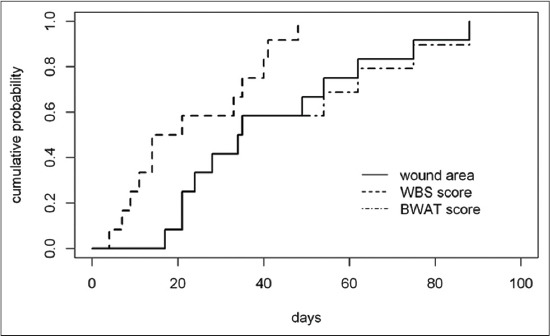
Cumulative probability to be healed considering complete wound area reduction and to reach the best Bates-Jensen Wound Assessment Tool and wound bed scoring scores during time.

The median TWH was 34 days. The 0.25, 0.50, and 0.75 cumulative probability to be healed in this study was reached at 21, 34, and 54 days, respectively (Figure-3). No wounds were considered healed at 14 days, 3 were considered healed within 21 days (cumulative probability of healing: 0.25; 95% CI: 0-0.46), 5 wounds within 28 days (cumulative probability of healing: 0.42; 95% CI: 0.06-0.64), 7 wounds within 35 days (cumulative probability of healing 0.58; 95% CI: 0.19-0.79), and 10 wounds within 63 days (cumulative probability of healing 0.83; 95% CI: 0.41-0.95) (Table-1).

In all wounds the best BWAT score was achieved at the same date when clinician assessed the complete healing. Eleven out of 12 wounds achieved the minimum BWAT score of 9 while 1 wound achieved the score of 10. The pattern of cumulative probability to achieve the best BWAT score was superimposable to that previously reported for wound area reduction (Figure-3).

Considering the WBS scale, the 0.25, 0.50, and 0.75 cumulative probability to achieve the best WBS score was reached at 9, 14, and 35 days, respectively (Figure-3). Two wounds reached the best WBS score within 7 days (cumulative probability 0.17; 95% CI: 0.00-0.35), 6 wounds reached the best WBS score within 14 days (cumulative probability 0.50; 95% CI: 0.12-0.72), 7 wounds reached the best WBS score within 21 days (cumulative probability 0.58; 95% CI: 0.19-0.79) and no wound further reached the best WBS score between 21 and 28 days. In all cases, the best score was reconfirm in the subsequent assessments until complete epithelialization was achieved.

**Figure-4:**
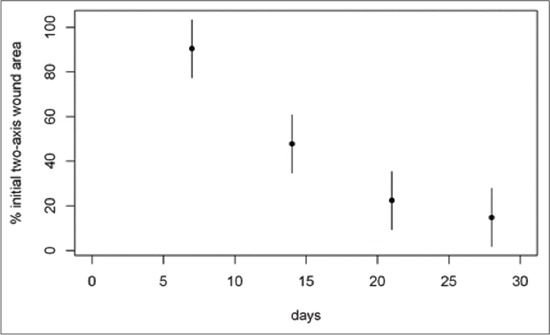
Mean percentage of “two-axis” wound area respect to T0 during the 1^st^ month of treatment. The mean percentage of wound area at day 7 was 90.41% (95% confidence interval [CI]: 77.38-103.4), at day 14 was 47.74% (95% CI: 34.72-60.8), at day 21 was 22.38% (95% CI: 9.36-35.4) and at day 28 was 14.81% (95% CI: 1.78-27.8) of the original size.

**Figure-5:**
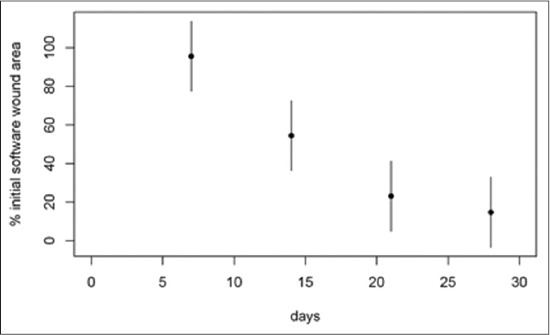
Mean percentage of software wound area respect to T0 during the 1^st^ month of treatment. The mean percentage of wound area at day 7 was 95.5% (95% confidence interval [CI]: 77.33-113.8), at day 14 was 54.4% (95% CI: 36.22-72.6), at day 21 was 23.10% (95% CI: 4.89-41.3) and at day 28 was 14.79% (95% CI: −3.42-33) of the original size.

**Figure-6:**
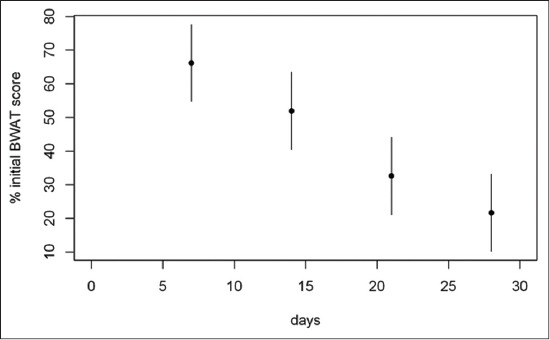
Mean percentage of initial Bates-Jensen Wound Assessment Tool score (BWAT) during the 1^st^ month of treatment. The mean percentage of BWAT score at day 7 was 66.15% (95% confidence interval [CI]: 54.7-77.6), at day 14 was 51.96% (95% CI: 40.5-63.4), at day 21 was 32.63% (95% CI: 21.1-44.1) and at day 28 was 21.70% (95% CI: 10.2-33.2) of the admission score.

**Figure-7:**
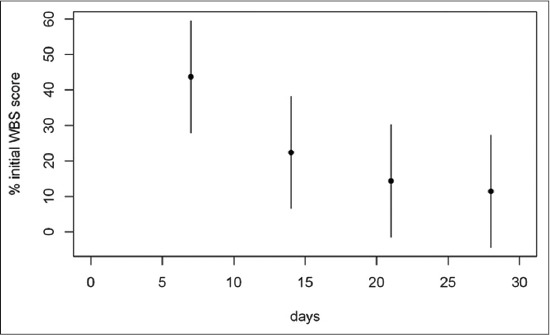
Mean percentage of initial wound bed scoring (WBS) score during the first month of treatment. The mean percentage of WBS score at day 7 was 47.71% (95% confidence interval [CI]: 27.86-59.6), at day 14 was 22.38% (95% CI: 6.52-38.2), at day 21 was 14.38% (95% CI: 0-30.2) and at day 28 was 11.43% (95% CI: 0-27.3) of the admission score.

Considering the 1^st^ month of treatment, the weekly mean percentages of two-axis wound area, software wound area, BWAT and WBS scores respect to admission values are illustrated in Figures-4-7, respectively.

When another clinician applied both scoring systems, the agreement between the two operators was considered good with an ICC of 0.88 for WBS (95% CI: 0.83-0.91) and of 0.95 for BWAT (95% CI: 0.93-0.97).

**Figure-8:**
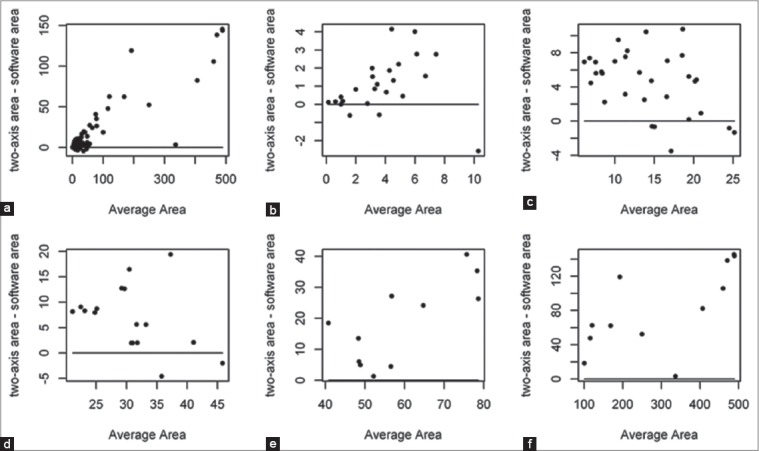
Comparison of area measurement between two-axis and software methods. Panel A: Overall wound area measures; Panel B: Two-axis wound area measures <10 cm^2^; Panel C: Two-axis wound area measures ≥10 cm^2^ and <25 cm^2^; Panel D: Two-axis wound area measures ≥25 cm^2^ and <50 cm^2^; Panel E: Two-axis wound area measures ≥50 cm^2^ and <100 cm^2^; Panel F: Two-axis wound area measures ≥100 cm^2^. An increasing trend for the discrepancy between the two methods with the increasing of the wound area is shown.

Regarding the comparison between area measurement with rectangular and software method, overall 107 measurements were performed with both the methods. Since 12 measurements for both method reported 0% at the time of complete healing, only the other 95 remaining measures were considered. As the differences between methods were related to the area, to facilitate the comparison data were subdivided according to the following categories: Areas <10 cm^2^ (28.4% of the data), areas between 10 and 25 cm^2^ (29.5% of the data), areas between 25 and 50 cm^2^ (17.9% of the data), areas between 50 and 100 cm^2^ (11.6% of the data) and areas beyond 100 cm^2^ (12.6% of the data). Measurements with rectangular approximation tend to be greater than measurements with software and the differences between the two measurements tends to increase with the increasing of the area (Figure-8).

## Discussion

The roles of HA in each phase of wound healing have been investigated intensively in human medicine, and HA’s properties have recently been successfully used in a number of wound dressing [28,36]. In veterinary medicine the potential for using HA-based medication on open skin wound in dogs was studied only by two case-control papers in which a cross-linked HA was applied on small (2 cm × 2 cm) experimental open wounds. In those studies controversial results were obtained and also taking into account the positive experience of human medicine, the additional investigation should be reached to increase the knowledge of HA properties also for companion animals [8,9]. In addition, medical experimental research does not always replicate clinical conditions [36] such as the presence of comorbidities or peripheral tissue damage that impair the healing process as happen instead in all non-experimental wounds making those results’ studies potentially no reproducible in the clinical setting. Regarding these issues, the use of HA-containing dressings was evaluated for the first time on a small cohort of non-experimental cutaneous open wounds in client-owned dogs.

The commercial available HA formulations tested in this study were found easy to apply, without the need of intensive staff training and so could be used not only in a veterinary second opinion hospital but even in an ambulatory setting. The formulations in gauze, cream, granules and spray allowed to apply the medication also in the presence of very irregular shape and inside the undermined pocket. The bandages were applied as routine so dogs were able to stay at home and to live a regular life with the exception of takes care of bandages (e.g. T-shirt covering the bandage, Elizabethan collar). Owners were instructed to re-build the bandages only when the wounds were in the late stage of progression, and no problems were recorded at the weekly examination using this strategy. In fact, the formulations in cream and spray were also described for auto-medication for human beings.

No side effects on wounds and periwounds tissue were recorded in the present study. This finding is in according with the past literature in which no side effect and no allergic response were reported for the topical application of HA in wounds, both for dogs and human [8,9,22,37,38]. Hadley and colleagues histologically proved the absence of inflammatory reaction around the HA-based gel applied on their experimental wounds in dogs [9]. The unpleasant odor reported by some owners associated with the HA products used in the present study was otherwise tolerated and has been related to the degradation of HA associated to the presence of wound fluid. To bypass the uncomfortable sensation linked to the use of spray, owners were instructed to not use it directly on the wound but to drench sterile gauze and to apply it on the wound.

The frequency between each dressing change varied widely and was mostly influenced by type of dressing and characteristic of the wound at assessment. The adsorbent medication made of partial benzyl ester derivative of hyaluronan was re-applied every about 4 days even if for human medicine this product could be leave until for a week. This medication breaks down in contact with wound exudate forming a gel rich of HA. The degradation timing depends on exudate levels and when at the assessment wound achieved a high exudate score the dressing change was scheduled more frequent than weekly. At the same time clinician had to consider this degradation and gel forming when evaluated the type and amount of exudate, especially when the bandage was early changed and the product was not totally adsorbed. It may be possible that this consideration has led to a more frequent dressing change in the first wounds treated respect to the last when a good mastery of the product was achieved.

One factor that should be taken into account in the choice of dressing is the cost benefits and even, for veterinary practitioners, the easy availability also for not hospital facility. The HA-containing dressings used in this study are available in any drug store of the country and have a cost range from of about 30 (gauze, 10 cm × 10 cm) to 10 euro (15 g cream tube) at which should be added the regular cost for bandage materials and clinical control. Obviously, the cost increases with increasing size of treated area and with the need of more than one product. On the contrary, the increased cost of an advance medical dressing has to be set against a shorter period of treatment and also to the lack of need of hospitalization for the type of medications used in this study.

In front of these good results is mandatory to achieve also a good wound outcome. To test the outcome of a wound medication a control group is always necessary. The explorative intent of this study, the small number of the sample and the difficult to build a control group that overlaps the characteristic of non-experimental wounds and patients allow to extrapolate only observational data. Authors considered the TWH (of about 1 month) achieved with HA-based dressing in canine non-experimental open wound a promising result. In particular, the wound area reduction was higher between 7 and 14 days with a mean wound area at 2 weeks halved than admission (Figures-4 and -5). Recently, the use of negative pressure in spontaneous wounds healed only by the second intention achieved a mean healing time of 26 days while excisional wound without advance dressing achieved a median healing time of 53 days [5,7]. Nevertheless due to the specific nature and features of spontaneous injuries any comparison with other studies that used different dressing or therapy involves possible high-risk bias and confirmation in further studies of the impact of the HA-medications on wound outcome is necessary.

Reading the literature on HA and wound healing seemed that HA plays a role in each phases of the healing process. Many studies found that HA not only enhance the fibroblast and vascular deposition in the granulation tissue, but also the keratinocyte proliferation during epithelialization [17-21]. Due to ethical implication and the clinical nature of this study no incisional biopsy of the healing tissues was sample during the present study, so was impossible to better elucidate the structure of the newborn tissues, and in addition in absence of a control group to have a comparison with no treated wounds. Otherwise, to authors’ experience and observation the granulation tissue filled the wound gap in a very quick period and generally grossly appeared of a very good and healthy quality making supposed that the HA have principally promoted the deposition of fibroblast and neo-angiogenesis. This is also reported by another experimental study on HA in dogs, in which authors described a better gross appearance of the granulation tissue for the treated wounds [8].

Periodic wound assessment to document healing progression and effectiveness of treatment is one of the most important parts of wound management. Wounds were reassessed at every bandage change. In human medicine, the frequency of assessment is often related to specific patient and wound, with a higher frequency for acute than chronic wound [29]. In veterinary experimental research with HA the wounds were re-assessed every 3 days or weekly [8,9] but no information on clinical setting is available. In author’s experience with these HA-based products the assessment may be generally repeated after a period from 4 to 7 days to detected noticeable alteration that justify to declare an improvement or worsening of the wound even if the clinician as to change early the dressing as happen in case of dogs prone to remove it.

Wound dimensions are the most frequent used parameters for healing evaluation in clinical and research settings [8-10,13,27]. Many are the possible measures to evaluate a dimensional assessment [39]. In veterinary medicine, the majority of the studies referred to area for measuring the wound extension [8-10,13]. In experimental setting often digital imaging and software application have been used [8-10] while linear metric dimension and mathematical formula have been used in clinical research [5]. Obviously, the technique to calculate the wound area as a rectangular only approximates the real wound area that could be of irregular shape, but it was made because was the simplest, least expensive, reliable and fast method in a clinical setting [39]. On the other hand, this could have overestimated the real size of about 25% in case of circular wound area [39]. In fact, the two-axis methods generally represent a bigger wound area respect to software assessment also in this present study. However, this method was always applied, and the possible error was repeated during the assessments making probably correct the comparison of two evaluations in the same wound. To be more precise even the software was applied. This software was free, easy to use and the only necessary equipment is a digital camera and a personal computer. However, the authors find some possible bias use it when the extension of the wound followed the natural curves of the body (e.g. wound extended for more than 180° around extremity) and the bi-dimensional picture achieved was not able to represent the real extension. In these cases, the technique reported by Tambella *et al*. where the perimeter of the open wound area was captured by hand with a fine tip permanent marking pen on a transparent sheet placed directly on the wound bed and then photographed and measured with the software should be use [14]. Taking in consideration the difference achieved with this two methods (Table-3) and the absence of a gold standard, the method chooses at the first assessment should be repeated during the healing process, at least until the wound became small (<9 cm^2^) and the difference could be more probably <1 cm^2^.

**Table-3:** Comparison between “two-axis” and “software” methods for measuring the wound area.

	Two-axis wound area <9 cm^2^ (%)	Two-axis wound area >9 cm^2^ and <25 cm^2^ (%)	Two-axis wound area >25 cm^2^ and <50 cm^2^ (%)	Two-axis wound area >50 cm^2^ and <100 cm^2^ (%)	Two-axis wound area >100 cm^2^ (%)
Total number of measurements	24	31	17	11	12
Difference between the two methods					
<1 cm^2^	12 (50)	5 (16)	-	-	-
≥1 cm^2^ and<2 cm^2^	6 (25)	1 (3)	2 (11.8)	1 (9.1)	-
≥2 cm^2^ and<3 cm^2^	6 (25)	3 (9.7)	3 (17.6)	-	-
≥3 cm^2^ and<4 cm^2^	-	2 (6.5)	-	-	1 (8.4)
≥4 cm^2^ and<5 cm^2^	-	4 (12.9)	1 (5.9)	2 (18.2)	-
≥5 cm^2^	-	16 (5.6)	11 (64.7)	8 (72.7)	11 (91.6)

Difference >1 cm^2^ were found in 50%, 84%, and 100% of the areas within 10 cm^2^, between 10 and 25 cm^2^ and >25 cm^2^ respectively. Differences >2 cm^2^ were found in 25%, 81%, 88%, 91% and 100% of the areas within 10 cm^2^, between 10 and 25 cm^2^, between 50 and 100 cm^2^ and >100 cm^2^, respectively. Differences >5 cm^2^ were found in 52%, 65%, 73% and 92% of the areas between 10 and 25 cm^2^, between 25 and 50 cm^2^, between 50 and 100 cm^2^ and >100 cm^2^, respectively

The use of wound area alone in the assessment of wound healing progression has the disadvantage to do not take into account the change in the quality of the wound bed that not always reflect a change or a reduction in wound dimensions [27]. In veterinary medicine no data are available concerning this kind of assessment even if some authors discussed about the addition in wound judgment of other parameters [14], such as fluid characteristics, granulation tissue, periwound, status and hidratation to subjectively evaluated experimental wound in dogs [9,13]. Considering this, two visual wound scales previously used in human medicine were parallel applied at each assessment. In human medicine this kind of evaluations were very diffuse and even if a real gold standard method is not approved, these tools represent an active part of wound management [27,35,40]. These scales are generally used and drawn for the evaluation of the most frequent human chronic wounds and their application on an acute open wound in dogs remained an explorative attempt.

In addition, the weight of the different tool characterizing the wound at the assessment reflect the mutability of the healing process and is unequivocally linked to the selection of appropriate dressing that should be based on the type of wound, healing status, bio-burden present and amount of exudate [35,41]. In this scenario, wound quality assessment is also performed to determine if wound is changing, not only because it actually may be healing, but also because changes provide useful tool to improve the plan of care and direct clinical decision-making [31]. In this case series the scores, in association with manufacture’s instruction linked to TIME approach, were used to direct decision about which HA-containing dressing should be apply. The TIME approach is based on the goal to remove local barriers to healing and optimizes the tissue environment to achieve wound healing. This concept of wound bed preparation in human medicine initially arose out of a need to address chronic wound but many wounds that fall into the traditional classification of “acute” could be amenable to management with the wound bed preparation concept [35]. The TIME concept of human medicine, although not specifically studied in the veterinary field, partially overlaps wound care guidelines reported for animal and are also reported in animal wound care book [1,42].

The best BWAT score for all wounds was obtained the same date of the total epithelialization while the best WBS score was achieved before. This result is justified by the fact that WBS score was delineated in human chronic wound to predict ultimate wound closure and not to assess the completion of the process as BWAT does, instead [34]. After the obtainment of the best WBS score, all the 12 wounds progressively healed until complete epithelialization. In addition using both scales, the greatest reduction of the score was obtained in the 1^st^ week with a mean decrease of 66.3% for WBS and 33.8% for BWAT respect to the admission scores. These results confirmed that qualitative assessment was especially useful in the early aspects of the healing of the acute wound in dogs when often the size of the wound do not change [27]. Furthermore, the wound that received debridement, even medical or surgical, resulted in a larger size that taking into account only this data could not revealed an improvement of the quality of the wound as instead confirmed by both scoring systems (Table-2).

In both scales many are the parameters considered to elaborate the final score. In human seems that exudate control, minimal eschar and decrease size have a higher prognostic impact on wound healing of chronic wound respect to other such as quality of granulation tissue [31,43]. The low number of cases in this study did not permit to analyze which parameters including in the scales could have the higher impact on second intention wound healing in acute wounds in dogs. The BWAT scale takes into account more parameters than the WBS and nowadays this could allow considering it more complete even taking into account the higher agreement achieved by two different operators. However, further studies are needed to delineate which parameters are more useful to describe the healing process in dogs and which are secondary or even unnecessary.

Based on these results, HA-containing dressings used in this study are easy to apply, well tolerated and carry promising results in improving healing of acute open wounds in dogs. In this explorative study the investigation of prognostic significances of site and size of the wound on healing time was not possible and further studies are necessary, also taking into account the comparison between different dressing and therapy on healing rate and on cost-benefit ratio. The use of scale scoring systems to assess wound healing gives consistent results complimentary to wound area reduction and the application of this assessment tools should be evaluated in veterinary medicine. The therapeutic approach based on quality wound assessment used in this study could be considered a primary pilot systemic decision-making process easy to follow wound treatment routine with HA-based dressing in daily veterinary clinical practice.

## Authors’ Contributions

RF, PB, and DS designed the study project. DS and RF retrieved all data during the study period. PB analyzed the data. The paper was written by RF. DS, SR, and GR have enrolled the cases and revised critically the paper. All authors read and approved the final manuscript.
